# Rapid Ag Nanofiber Formation Via Pt Nanoparticle-Assisted H_2_-Free Reduction of Ag^+^-Containing Polymers

**DOI:** 10.1186/s11671-021-03549-4

**Published:** 2021-05-26

**Authors:** Xu Zhao, Yukiko Kawamura, Mikio Muraoka

**Affiliations:** Department of Systems Design Engineering, Akita University, Akita, 010-8502 Japan

**Keywords:** Ag nanofibers, Aspect ratio, Percolation, Transparent conductive films

## Abstract

One-dimensional Ag nanostructure-based networks have garnered significant attention as next-generation transparent conductive materials. Ag nanofibers (NFs) with high aspect ratios decrease the number density required for percolation; hence, they form qualitatively superior transparent conductive films. This study reports a novel method for rapidly fabricating Ag NFs via Pt nanoparticle-assisted H_2_-free reduction of solid-state AgNO_3_. Our results first indicated that polymers can be a source of hydrogen gas in the presence of Pt nanoparticles; Ag NFs with aspect ratios above 10^5^ were obtained herein by heating AgNO_3_-containing polymer NFs in a short period of time and in an open-air environment. Our method not only successfully reduced the amount of polymer residue often encountered in spun NFs but also created an effective self-supporting reduction system that does not require an external reducing gas supply. The obtained Ag NF networks were highly conductive and transparent. Moreover, the mechanism of Ag NF formation was investigated. We demonstrate that the proposed method exhibits a high potential for producing high yields of Ag NFs in a simple and rapid manner.

**Supplementary Information:**

The online version contains supplementary material available at 10.1186/s11671-021-03549-4.

## Introduction

Transparent conductive films are widely used as transparent electrodes in liquid crystal displays, solar cells, smart windows, touch screens [1–6], transparent film heaters [7–11], and electromagnetic wave shielding materials [12–14]. The latest transparent conductive material, indium tin oxide (ITO), has outstanding conductivity and transparency in the visible region [15]. However, with the increasing demand for flexible conductive materials in recent years, the lack of flexibility of ITO and scarcity of In have prompted research into viable alternatives. Numerous candidates, such as carbon nanotubes [1, 7, 16], graphite [8, 17, 18], conducting polymers [19, 20], and metallic nanowires (NWs) [3–5, 9–11], have been extensively studied. In particular, Ag NW networks [3, 4, 9] appear to be promising alternatives. In addition to the excellent conductivity, stretchability, and flexibility derived from the metallic properties of Ag, a wire diameter smaller than the visible light wavelengths ensures high transparency of the network. Compared to ITO, Ag NW networks have the benefits of a wider wavelength range with exceedingly high transparency [21]. These properties can be applied to photovoltaic systems to improve the conversion efficiency of solar cells.

Currently, the polyol approach [22, 23] is the most promising route for synthesizing Ag NWs. The Ag NWs synthesized by this solution-based process can easily be dispersed to form a network. However, the contact points between the NWs strongly influence the properties of the networks. The high contact resistance greatly increases the sheet resistance, while weak bonding worsens the mechanical properties when the network is deformed. Previous studies have indicated that longer NWs could yield qualitatively better transparent conductive films because doubling the length of the NWs decreases the number density required for percolation by a factor of four [24]. Nevertheless, existing synthesis methods have limited the length of Ag NWs to several tens of micrometers and the aspect ratios to 10^2^–10^3^; hence, the issues caused by the contact points remain a challenge.

Compared to Ag NWs, Ag nanofibers (NFs) are approximately the same size in diameter, but they are much longer (usually several tens of millimeters) and have higher aspect ratios that can reach 10^5^–10^6^. However, there are few reports on the synthesis of Ag NFs. Although Ag^+^-containing precursor NFs can be mass-produced through electrospinning [2] and blow-spinning [25], the challenges faced in this synthesis include the reduction of Ag^+^ to form continuous Ag NFs and the decomposition of the residual insulating polymers originating from the precursor solution. Recently, Lin et al. reported a method for reducing silver nitrate (AgNO_3_) NFs by UV irradiation [6]. A large-scale Ag NF network was obtained after 3 h of UV irradiation to reduce Ag^+^. Nevertheless, the reduction process was relatively long, and the decomposition of residual polymers remained an issue. However, the catalytic effect of metal nanoparticles [26, 27] has given us the inspiration that polymers may be effectively used in the presence of specific metal nanoparticles.

This study reports a simple method for the fabrication of Ag NFs. Our findings indicate that polymers can be a source of hydrogen gas in the presence of Pt nanoparticles; we obtained Ag NFs with high aspect ratios by heating AgNO_3_-containing polymer NFs. The resultant Ag NF networks were highly conductive and transparent. This proposed method has high potential for producing high yields of Ag NFs in a simple and rapid manner.

## Experimental

**Fig. 1:**
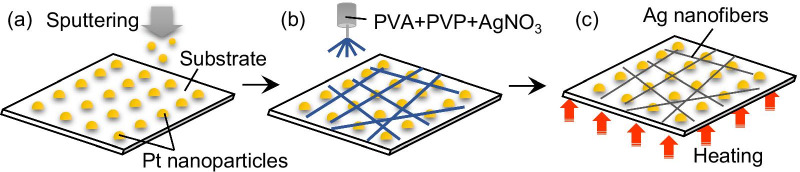
Schematic showing the Ag NF network fabrication process: **a** Pt sputtering, **b** electrospinning of the AgNO_3_/PVA/PVP-mixed NF network, and **c** heating in air

The experimental procedure for the fabrication of the Ag NF networks is illustrated in Fig. 1. Pt nanoparticles were deposited on an 18 × 18 mm^2^ micro cover glass substrate with a thickness of 120–170 µm using a magnetron sputtering system (SC-701HMCII, SANYU ELECTRON Co., Ltd.) at 23 °C (Fig. 1a). The purity of the Pt target was 99.99%. The deposition pressure and rate were 1.5 Pa and 2.5 Å/s at 25 mA, respectively, and were determined in order to obtain a homogeneous distribution of nanoparticles in a precise quantity. The deposition time was 4 s, after which the thickness of the deposited Pt was 1 nm. Notably, this thickness did not result in a continuous Pt film but discontinuous islands (nanoparticles). This phenomenon is known as the initial stage of thin metal film growth [28-30]. These Pt nanoparticles are crucial in the fabrication of Ag NFs, as discussed in detail in later sections. After the deposition of the Pt nanoparticles, an electrospun AgNO_3_/polyvinyl alcohol (PVA)/polyvinyl pyrrolidone (PVP)-mixed NF network was deposited onto the substrate by applying 20 kV voltage to the AgNO_3_/PVA/PVP aqueous solution for 5 min at a collection distance of 15 cm (Fig. 1b). A syringe with a needle of 0.41 mm inner diameter was connected to a micropump. The flow rate of the micropump was set to 0.05 mL/h. The pumped solution was composed of AgNO_3_ (powder, purity 99.8%), PVA (polymerization degree: 1500), PVP, and deionized water in a weight ratio of 10:8.5:4:100 in wt.%. The viscosity of the solution was 277 mPa s. The molecular weights of PVA and PVP are 6.6 × 10^4^ g/mol and 4 × 10^4^ g/mol, respectively. PVA is a common polymer material used for electrospinning, while PVP is used as a molecular capping agent. Finally, the specimens were heated in air at 250 °C for 30 min to reduce AgNO_3_ to Ag in the presence of Pt nanoparticles (Fig. 1c). The products were measured using the four-probe method and analyzed by atomic force microscopy (AFM: Dimension Icon, Bruker Japan Co., Ltd.), X-ray diffraction (XRD: Smart Lab, Rigaku Co., Ltd.), field-emission scanning electron microscopy (FE-SEM: SU-70, HITACHI Co., Ltd.), high-angle annular dark field scanning transmission electron microscopy (HAADF-STEM: Talos F200X, FEI Co., Ltd.), energy dispersive X-ray (EDX), and Raman spectrometry (RAMANtouch, Nanophoton Co., Ltd.).

## Results and Discussion

**Fig. 2:**
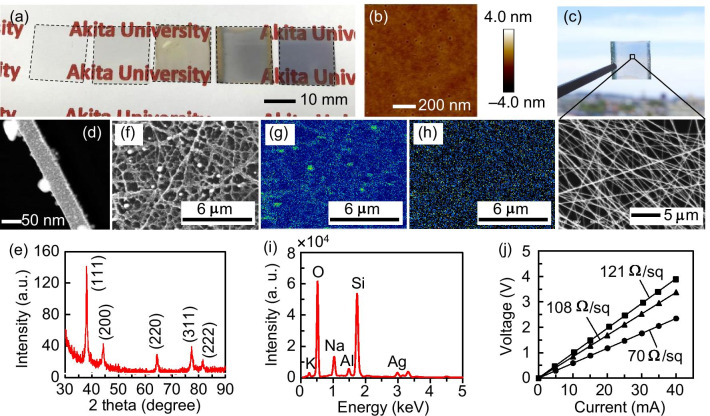
**a** States of the specimens during the experiment. **b** AFM image of Pt sputtered on a glass substrate. **c** Optical and FE-SEM images of the specimen after heating. **d** HAADF-STEM image of Ag NF. **e** XRD pattern of Ag NF. EDX analysis results of the Ag NF network: **f** SEM image, **g** Ag, and **h** C distribution mappings, and **i** qualitative analysis of the area shown in (**f**). **j** Current–voltage curves of the specimens measured using the four-probe method

Figure 2a shows the states of the specimens during the experiment. Each specimen is marked with a dashed line. The illustrations from left to right are of the prepared glass substrate, the glass after Pt sputtering, the NF network after electrospinning, the sample after heating (the yellow portions are Au electrodes in size of 18 × 1.5 mm^2^ used for resistance measurement), and a 15-nm-thick Ag film deposited on a glass substrate for reference. Figure 2b shows an AFM image of Pt deposited at 2.5 Å/s for 8 s on a glass substrate. The formation of a discontinuous film having a rough surface and containing a large number of small holes with a diameter of 10–20 nm and a depth of 2–3 nm was confirmed. The depth was highly consistent with the desired film thickness. The Pt nanoparticles were distributed in clusters with an average in-plane size of 32 nm. This may be because metals with high boiling points have high values of supersaturation and small critical nuclei, and they easily condense [31]. We believe that the Pt nanoparticles are distributed in a more dispersed state when the film thickness is 1 nm. After heating, the specimen displays high transparency in the visible region (Fig. 2c). The long NF-percolated network is clearly illustrated in the magnified view of the SEM micrograph (Fig. 2c). The area fraction of the network, measured by applying thresholds to the SEM micrograph using image analysis software (WinROOF2015, MITANI Corporation), is approximately 47%. The HAADF-STEM analysis (Fig. 2d) demonstrates that the NFs were several tens of nanometers in diameter and had a polycrystalline microstructure. The electrospun NFs spanned the substrate; hence, their length was approximately 18 mm or even longer. Therefore, the aspect ratio of the present NFs reached an order of 10^5^ or even larger. All the peaks in the XRD pattern (Fig. 2e) are in good agreement with those of the face-centered cubic structure of Ag, indicating that Ag NFs were successfully obtained and finely crystallized. The EDX analysis results (Fig. 2f–i) indicate that the network was composed of Ag NFs, with no C-related distribution in the NFs. Moreover, Pt was not detected, probably because it was present in only a small amount. The detected Si, O, Na, Al, and K elements (Fig. 2i) originated from the glass substrate and thus can be ignored. The measured current–voltage curves (Fig. 2j) demonstrate that the formed Ag NF networks have metallic properties, and their sheet resistances are as low as several tens of Ω/sq, which is comparable to that of commercially available ITOs. The present Ag NF network can be easily applied to a film substrate as a flexible transparent electrode (e.g., see Additional file 1: Fig. S1).

As a result, the role of Pt nanoparticles and the importance of heating in air, instead of under vacuum, were questioned. Therefore, these aspects are discussed in the following paragraphs.

The initial purpose of Pt nanoparticle deposition was to improve the electrospinning process so that more AgNO_3_/PVA/PVP NFs could be deposited on the insulated glass substrate. Interestingly, when we heated the AgNO_3_/PVA/PVP NFs with Pt nanoparticles at 250 °C, we obtained Ag NFs. The XRD and EDX analysis results (Fig. 2e–i) strongly support this novel finding. It is highly improbable that Ag was produced by the thermal decomposition of AgNO_3_ after heating at 250 °C because the decomposition of AgNO_3_ occurs at temperatures above 500 °C [32]. Therefore, it is more likely that Ag was produced from the hydrogen reduction of AgNO_3_. Moreover, the hydrogen gas most likely came from PVA and PVP used in the experiments. However, to date, there have been no reports that hydrogen gas can be directly generated from the thermal decomposition of PVA or PVP. Most reports have indicated that the main decomposition product of PVA at approximately 200 °C is H_2_O [33-36]. We postulate that Pt nanoparticles are the decisive factor for the reduction process. Pt has been the subject of much research and is well known as a catalyst for chemical reactions.

The above discussion is summarized by Eqs. (1) and (2) given below.

1 $${\left[{\mathrm{CH}}_{2}\mathrm{CHOH}\right]}_{n}\begin{array}{c}\stackrel{\mathrm{ Pt }}{\to }\\ {\mathrm{ in~air}}\end{array}{\mathrm{CO}}_{2}+{\mathrm{H}}_{2}+{\mathrm{H}}_{2}\mathrm{O}$$

and

2 $${\mathrm{H}}_{2}+{\mathrm{Ag}}^{+}\to \mathrm{Ag}+{\mathrm{H}}^{+}.$$

The main components of PVA ([CH_2_CHOH]_*n*_) and PVP ([C_6_H_9_NO]_*n*_) are similar, and PVA accounts for the majority of the electrospinning solution; therefore, we focus on PVA in the following discussion. The reactions shown in Eqs. (1) and (2) were proposed based on our speculation because it is significantly challenging to prove that PVA produces hydrogen gas via thermal decomposition in the presence of Pt nanoparticles. However, according to our experimental results and other comprehensive considerations, these reactions seem most likely to have occurred. To verify the catalytic effect of the Pt nanoparticles, other metal nanoparticles were also deposited (similar to the process shown in Fig. 1a), and the experiments were repeated. As shown in Table 1, Ag and Au nanoparticles were deposited onto glass substrates. Their thicknesses were restricted to 1 nm, which is a similar size to that of the Pt nanoparticles. Glass substrates without any nanoparticle deposition were also prepared for comparison. To ensure the reproducibility of the experimental results, at least four test pieces were prepared for each type of metal nanoparticle. These test pieces were then subjected to electrospinning (Fig. 1b) and heating (Fig. 1c) under the same conditions as those used for the Pt nanoparticles. Comparing the sheet resistances before and after heating demonstrates that only the sheet resistances of the test pieces with Pt nanoparticles were greatly reduced from being insulated to measuring several tens to hundreds of Ω/sq. This result implies that the AgNO_3_ component changed to Ag. Therefore, we conclude that Pt nanoparticles play a critical role in the successful fabrication of Ag NFs. Due to the presence of Pt nanoparticles, the insulating polymer materials (PVA and PVP) did not only thermally decompose but also effectively produced hydrogen gas that could reduce AgNO_3_.

**Table 1:** Sheet resistance variations of the test pieces with different metal nanoparticles

Nanoparticles	Heating conditions	Sheet resistances of the test pieces (Ω/sq)	
		Before heating	After heating
Pt	250 °C, 30 min	Insulated	Several tens to hundreds
Ag	250 °C, 30 min	Insulated	Insulated
Au	250 °C, 30 min	Insulated	Insulated
None	250 °C, 30 min	Insulated	Insulated

**Fig. 3:**
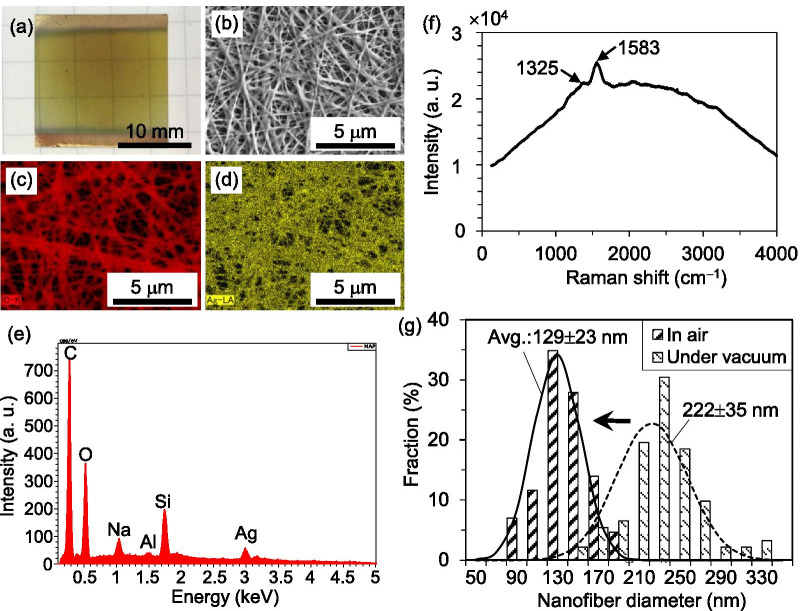
Specimen after heating under vacuum with **a** optical image and EDX analysis results: **b** SEM, **c** C, and **d** Ag distribution mappings; **e** qualitative analysis of the area shown in (**b**); and **f** the Raman spectrum. **g** Histograms of the NF diameter after heating in air and under vacuum

Reduction of metallic ions using reducing gases is usually carried out under vacuum. Therefore, the size of the specimen is limited by the vacuum chamber, and a significant amount of time is spent creating the vacuum. Fortunately, our method does not require a vacuum environment because we found that an open-air environment is more suitable for fabricating highly conductive Ag NFs. For example, Fig. 3a shows a specimen (prepared by the same processes shown in Fig. 1a, b) after heating under vacuum (using a flow rate of 200 sccm Ar gas and a pressure of 130 Pa at 250 °C for 30 min). Unlike the specimens heated in air, the pieces heated under vacuum were translucent and light brown in color. Remarkably, their sheet resistances were as high as several thousand Ω/sq, which is one to two orders of magnitude greater than those heated in air. The EDX analysis results (Fig. 3b–e) indicate that a significant amount of C is present in the NFs, while the detected Si, O, Na, and Al (Fig. 3e) came from the glass substrate and thus were ignored. Raman spectroscopic analysis (Fig. 3f) demonstrates that these carbon atoms have an amorphous structure. Two characteristic Raman peaks at approximately 1325 and 1583 cm^−1^ were detected, which are consistent with the peaks reported elsewhere [6]. Furthermore, as shown in Fig. 3f, an increase in the baseline of the Raman spectrum was detected due to fluorescence, which implies a high possibility of residual organics (polymers). Therefore, the test pieces heated under vacuum showed very large sheet resistances.

Figure 3g displays the histograms of the NF diameter after heating in air and under vacuum. The diameters were measured using the measuring function of SEM. Under each heating condition, more than 40 NFs were randomly selected for the SEM observations (at 5000 × magnification) and subsequent measurements. Compared to those heated under vacuum, the average diameter of the NFs after heating in air is approximately 100 nm thinner. This may be the result of the oxidation of amorphous carbons and their release through the vapor phase (CO_2_). The removal of amorphous carbons from the NFs perhaps greatly reduced the sheet resistances. The above discussion can be explained by the following chemical reaction:

3 $${\left[{\mathrm{CH}}_{2}\mathrm{CHOH}\right]}_{n}\begin{array}{c}\stackrel{\mathrm{ Pt }}{\to }\\ {\mathrm{ in~vacuum}}\end{array}\mathrm{C}+{\mathrm{H}}_{2}+{\mathrm{H}}_{2}\mathrm{O}.$$

The preferred product of thermal PVA decomposition is H_2_O during heating under vacuum; hence, C cannot be oxidized by oxygen, and residual amorphous carbons are produced. In contrast, as expressed in Eq. (1), the presence of air during heating provides the oxygen needed for the oxidation of carbons. Therefore, an open-air environment is more suitable for fabricating highly conductive Ag NFs.

**Fig. 4:**
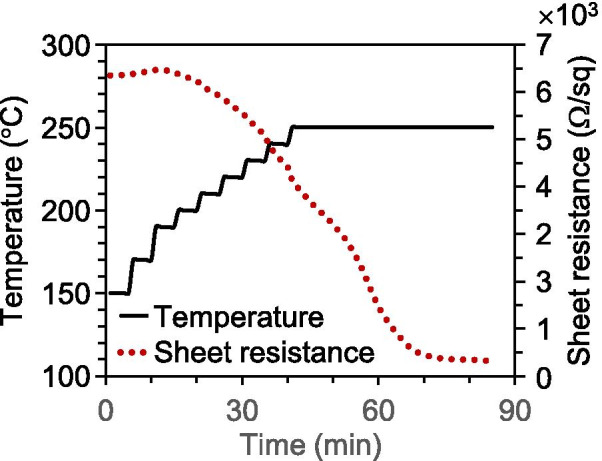
Variation in the sheet resistance with heating temperature and time

In addition to providing a simple fabrication method, we considered whether the proposed method could be more efficient and energy-saving. Figure 4 displays the in situ measurements of the sheet resistance. The specimen was preheated under vacuum (using a flow rate of 200 sccm Ar gas and pressure of 130 Pa at 250 °C for 30 min) so that its initial sheet resistance was approximately 6340 Ω/sq. The specimen was then heated from 150 to 250 °C in air. The profile of the heating temperature is represented in Fig. 4 by the solid line, while the sheet resistance is represented by the dotted line. The sheet resistance increased almost linearly as the specimen was heated from 150 to 200 °C because of the increase in the electrical resistivity as the temperature increased. Nevertheless, at 200 °C, the sheet resistance began to decrease rapidly, although the heating temperature continued to increase. After approximately 55 min, the sheet resistance dropped from 6420 Ω/sq to approximately 400 Ω/sq, and then the decreasing trend began to saturate. This phenomenon might have been caused by the oxidation of amorphous carbons and their release, as discussed above. Therefore, the heating temperature can be reduced to approximately 200 °C for the fabrication of Ag NFs. This progress is not only helpful for saving energy but also widens the range of options for heat-resistant substrates.

## Conclusions

In summary, a simple method for fabricating Ag NFs via Pt nanoparticle-assisted reduction of AgNO_3_ was proposed, and the mechanism was investigated. Although the method needs to be improved further, it has high potential to produce high yields of Ag NFs with high aspect ratios and transparent conductive films in a simple, rapid, and economical manner. Pt nanoparticles can be deposited onto a substrate by vacuum deposition or using a commercial Pt dispersion liquid. In theory, most silver salts, such as silver chloride, silver sulfide, and silver fluoride, can be reduced; therefore, the source of Ag^+^ is not limited to AgNO_3_. In addition, we predict that the other platinum group metals, such as Pd and Rh, may produce the same catalytic effect as Pt because of their similar chemical properties.

## Supplementary Information

**Additional file 1.** **Figure S1.** Electrical resistance of a flexible transparent electrode (a) without any deformation and (b) under bending deformation. The inset in (b) is a side view of the electrode attached to a glass tube with outer diameter of 20 mm; electrical resistance of a flexible transparent electrode (c) without any deformation and (d) under torsion deformation.

## Data Availability

All data generated or analyzed during this study are included in this article and its supplementary information file.

## Abbreviations

AFM: Atomic force microscopy
AgNO_3_: Silver nitrate
EDX: Energy dispersive X-ray
FE-SEM: Field-emission scanning electron microscopy
HAADF-STEM: High-angle annular dark field scanning transmission electron microscopy
ITO: Indium tin oxide
NW: Nanowire
NF: Nanofiber
PVA: Polyvinyl alcohol
PVP: Polyvinyl pyrrolidone
XRD: X-ray diffraction
