# Supplementary material for: Rapid Ag Nanofiber Formation Via Pt Nanoparticle-Assisted H2-Free Reduction of Ag+-Containing Polymers
Source: Nanoscale Res Lett. 2021 May 26;16:96. doi: 10.1186/s11671-021-03549-4 (PMC8155139; doi:10.1186/s11671-021-03549-4)
Supplement: Supplementary file 1 — Additional file 1. Figure S1. Electrical resistance of a flexible transparent electrode (a) without any deformation and (b) under bending deformation. The inset in (b) is a side view of the electrode attached to a glass tube with outer diameter of 20 mm; electrical resistance of a flexible transparent electrode (c) without any deformation and (d) under torsion deformation. [file 11671_2021_3549_MOESM1_ESM.docx]

**Supplementary Information**

**Rapid Ag nanofiber formation via Pt nanoparticle-assisted H_2_-free reduction of Ag^+^-containing polymers**

Xu Zhao^1,*^, Yukiko Kawamura^1^, Mikio Muraoka^1^

^1^Department of Systems Design Engineering, Akita University, Akita 010-8502, Japan

^*^Corresponding author. E-mail: zhao@gipc.akita-u.ac.jp

Figure S1 shows an example of applying Ag nanofiber (NF) network fabricated on a polyethylene naphthalate film substrate (thickness: 50 μm) as a flexible transparent electrode. The heating temperature used to reduce AgNO_3_ was 220 °C. The transparent electrode shown in Figs. S1a and S1b has a size of 26 × 20 mm^2^ and initial sheet resistance of 38 Ω/sq, while the one shown in Figs. S1c and S1d has a size of 40 × 40 mm^2^ and initial sheet resistance of 363 Ω/sq. These transparent electrodes show excellent flexibility. Even under severe bending and torsion deformation, there was no obvious change in electrical resistance.


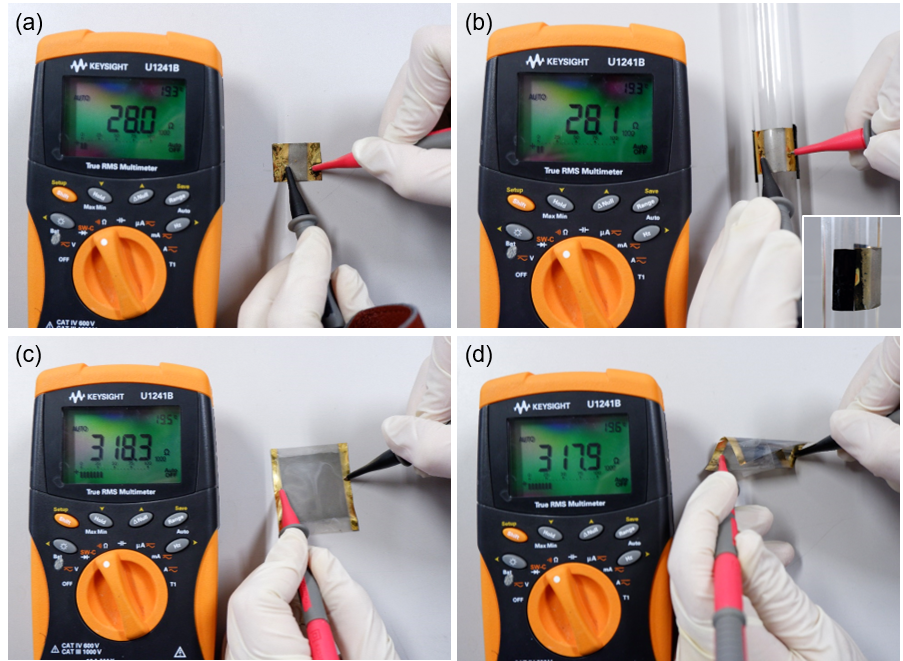


**Fig.S1** Electrical resistance of a flexible transparent electrode (a) without any deformation and (b) under bending deformation. The inset in (b) is a side view of the electrode attached to a glass tube with outer diameter of 20 mm; electrical resistance of a flexible transparent electrode (c) without any deformation and (d) under torsion deformation.
